# Qualitative analysis of acid washed black cumin seeds for decolorization of water through removal of highly intense dye methylene blue

**DOI:** 10.1016/j.dib.2018.08.096

**Published:** 2018-09-06

**Authors:** Sharf Ilahi Siddiqui, Geetanjali Rathi, Saif Ali Chaudhry

**Affiliations:** Department of Chemistry, Jamia Millia Islamia, New Delhi, India

**Keywords:** Water, Dye, Methylene blue, Removal, Adsorption, Black cumin

## Abstract

Dyes in water change the colour, taste and odour of water, are highly visible, and can be toxic and cancerous for the coloured water consumption human beings. Basic dyes particularly, methylene blue, MB has high colour intensity, shows intense colour even at low concentration, and are very toxic due to their complex structure. Instead of adsorption, removal of MB from water using various traditional treatment methods is costly and less effective. The use of bioadsorbent provides easy and low cost technique for removal of MB. For searching the adequate technique of dye removal, adsorption efficiency and mechanism of bioadsorbent can be analyzed. To this, MB removal efficiency of seeds of medicinal plant, black cumin seeds were analyzed. The data are supplied in the article.

**Specifications table**

**Table:** 

Subject area	Environmental Chemistry
More specific subject area	Adsorption
Type of data	Table, image, graph
How data was acquired	FTIR, XRD, SEM-EDX and TEM
Data format	Analyzed
Experimental factors	Hydrochloric acid washing of black cumin seeds, amount of black cumin seeds, initial concentration of methylene blue, time of reaction and temperature of reaction
Experimental features	Methylene blue removal efficiency of black cumin seeds
Data source location	New Delhi, India
Data accessibility	The data is with this article
Related research article	The Data are in “Sharf Ilahi Siddiqui, Geetanjali Rathi, Saif Ali Chaudhry, Acid washed black cumin seed powder preparation for adsorption of methylene blue dye from aqueous solution: Thermodynamic, kinetic and isotherm studies. J. Mol. Liq. 264, 2018, 275-284.

**Value of the data**

• Black cumin seeds are highly porous, amorphous and have large functional sites.
• High rate and efficiency of removal of methylene blue from water.
• Low quantity of black cumin seeds is sufficient.

## 1 Experimental design

**Scheme 1:**
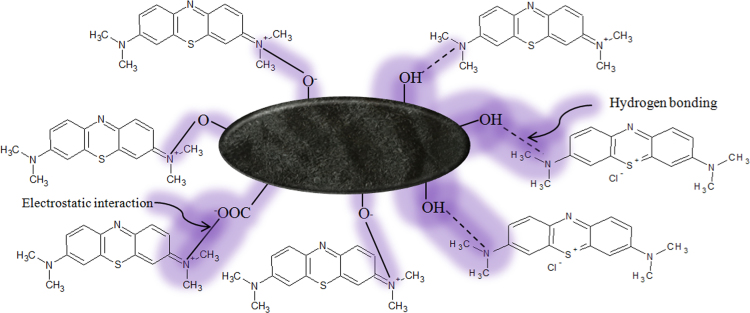
: Proposed mechanistic pathway for electrostatic and hydrogen bonding interactions between MB and AWBC.

Black cumin seeds were washed with inorganic acid, hydrochloric acid. Surface and particles properties of acid washed black cumin seeds were analyzed by FT-IR, XRD, SEM-EDX and TEM [1] (Supplementary Fig. S.1–5). MB removal efficiency of acid washed black cumin seeds, AWBC were analyzed according to the batch adsorption experiments under the various conditions such as effect of amount of AWBC, pH of solution, concentration of MB in solution, time and temperature of reaction [1] (Supplementary Fig. S.6–9). The efficacies of AWBC were compared to the un-washed black cumin seeds [1]. The concentrations of MB before adsorption and after adsorption in the water were analyzed by UV–vis absorption spectroscopy. The FT-IR spectrum of post adsorption AWBC ((Supplementary Fig. S.1) confirmed the interaction between AWBC and MB dye (Scheme 1) [1]. The removal efficiency of AWBC for MB was compared to other adsorbent (Table 1).

**Table 1:** Comparative MB removal study.

Bio-adsorbent	MB removal capacity (mg g^−1^)	Ref.
*Cortaderia selloana* flower spikes	40	[8]
*Phragmites australis*	22.7	[9]
Mesoporous silica	65.7	[10]
Hydrophobic silica aerogel	65.74	[11]
Hydrophilic silica aerogel	47.21	
ZnS:Ni-NP-AC	21.79	[12]
Cu(OH)_2_-NP-AC	32.9	[13]
Sunflower seed husk (Helianthus annuus)	4.76–23.20	[14]
Water hyacinth root powder	8.04	[15]
Dragon fruit peels	62.58	[16]
Raw algerian kaolin	52.76	[17]
Salix babylonica leaves powder	60.97	[18]
Spent yerba mate ilex paraguariensis	52.00	[19]
**Acid washed Black Cumin seed material**	**73.53**	**[1]**

## 2 Materials and methods

### 2.1 Washing of black cumin seeds

The washed, dried and grounded, seeds of black cumin were washed with common inorganic acid, Hydrochloric acid (HCl) as per the method reported literature [1], [2] to leach out the others elements attached on their surface.

### 2.2 Determination of surface properties of black cumin seeds

FT-IR spectrum analyzed for the functional groups present on the surface of AWBC which acted as adsorptive sites for MB molecules. The diffraction peaks in XRD pattern of AWBC were used to analyze the amorphous nature of the AWBC. SEM and TEM images are given for the porous and heterogeneous surface of AWBC, respectively. EDX pattern were analyzed for chemical composition of AWBC. The graph between ∆pH= pHi-pHf and pHi gave the zero point charge of AWBC [1].

### 2.3 Batch adsorption experiments

Batch adsorption experiments were carried out by agitating (at 215 rpm) the series of 50 mL of Erlenmeyer flasks having 10 mL of MB dye solution of an initial concentrations varying from 10 to 60 mg L^−1^ and 1 gL^−1^ of AWBC for contact time of 0–120 min at neutral pH, and room temperature. The concentration of MB in the solution before agitation and after agitation was estimated by analyzing their absorbance using ultraviolet-visible (UV–vis) spectrophotometer at 660 nm. These estimated initial, *C*_*o*_ and final concentrations, *C*_*e*_ of MB solution, respectively, gave the uptake capacity as follows [3], [4], [5]:

(1) $$MaximumuptakeofMB,Q_{e}=(C_{o}-C_{e})\frac{V}{m}$$

where, *V* is the volume of MB solution in liter and *m* (g) is the amount of AWBC.

(2) $$Percentageremoval,R\%=\left(\frac{C_{o}-C_{e}}{C_{o}}\right)100$$

Ultimately, adsorption data obtained from above study was verified by fitting in various isotherms, kinetic and thermodynamic relationships [6], [7] to design the appropriate water treatment system using bio-adsorbent [1].
