# Supplementary material for: Qualitative analysis of acid washed black cumin seeds for decolorization of water through removal of highly intense dye methylene blue
Source: Data Brief. 2018 Sep 6;20:1044–7. doi: 10.1016/j.dib.2018.08.096 (PMC6138838; doi:10.1016/j.dib.2018.08.096)
Supplement: Supplementary file 2 — Supplementary material [file mmc2.docx]

**Supplementary data**


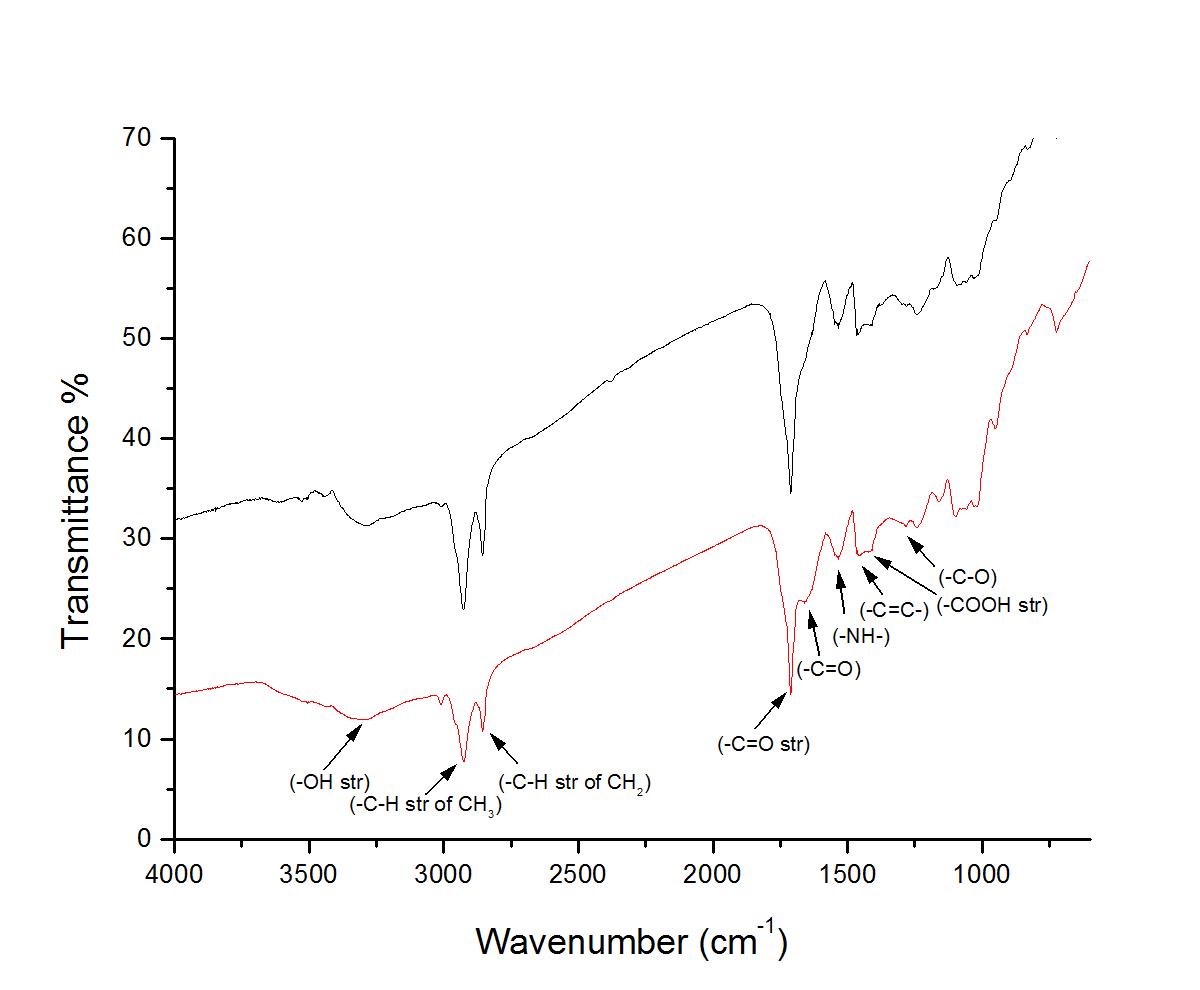


Fig. S1. FT-IR spectrum of AWBC (red) and MB loaded AWBC (black)


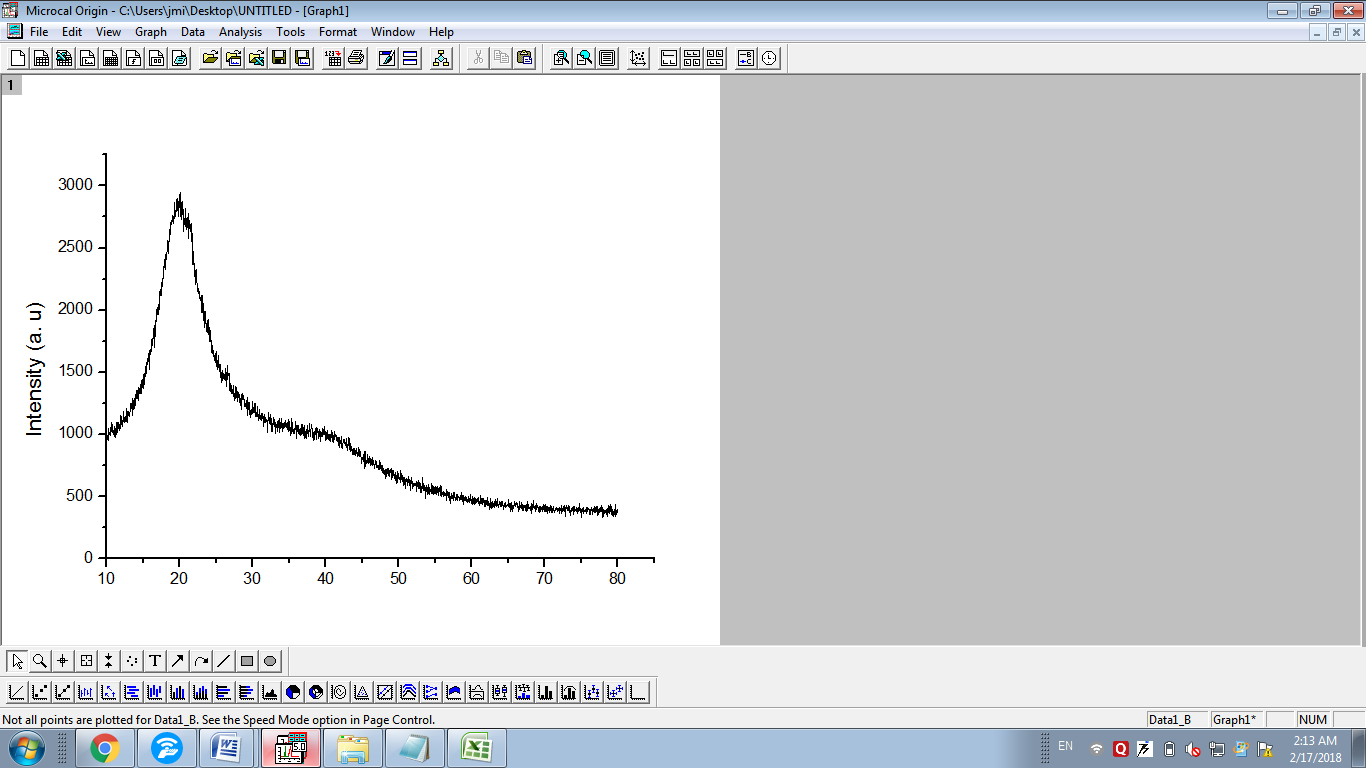


2θ

Intensity

Fig. S2. XRD pattern of AWBC


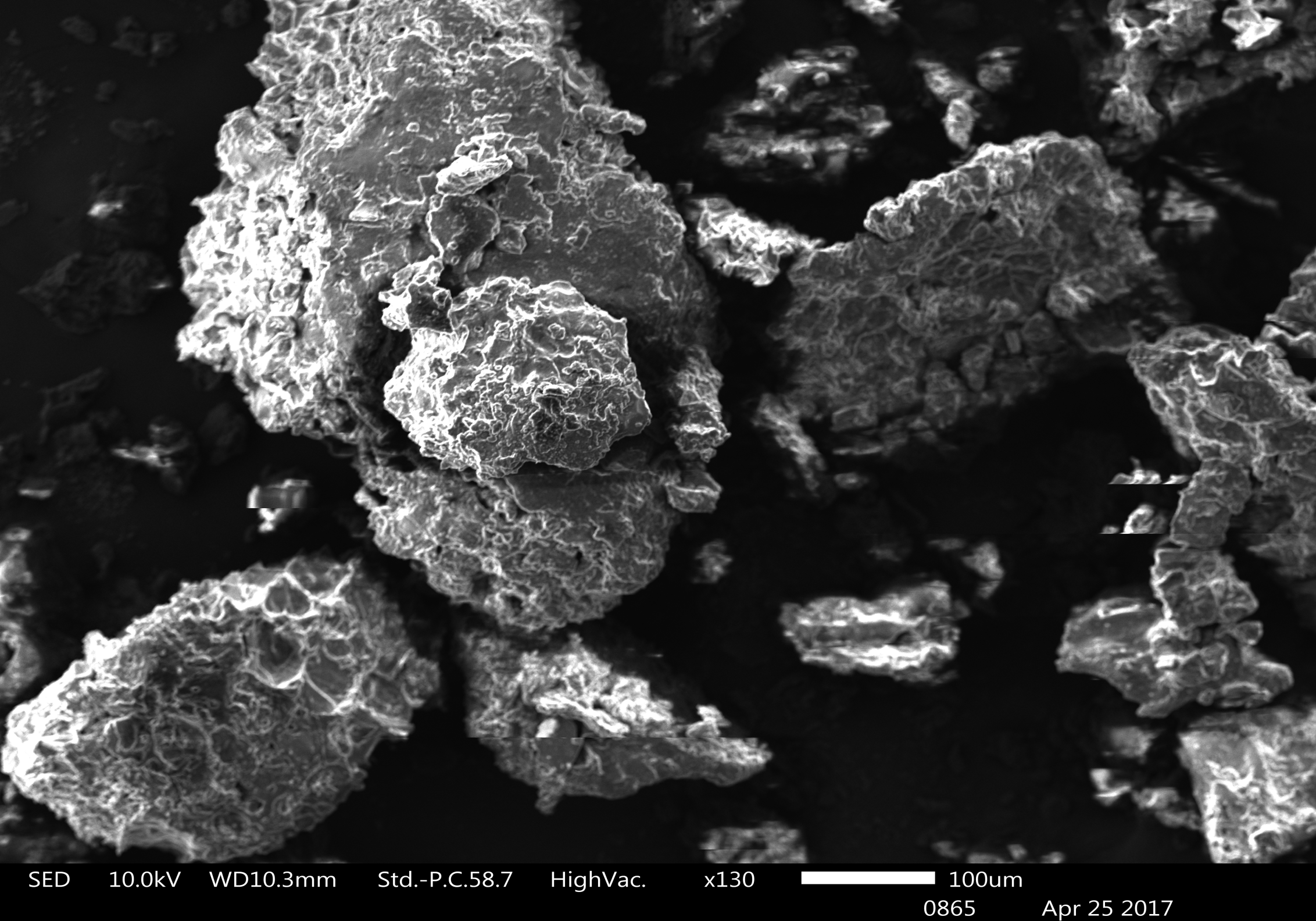


Fig. S3. SEM image of AWBC

| Element | [wt.%] | [norm. at.%] |
| --- | --- | --- |
| Carbon | 69.386 | 74.720 |
| Nitrogen | 04.618 | 04.265 |
| Oxygen | 25.995 | 21.015 |


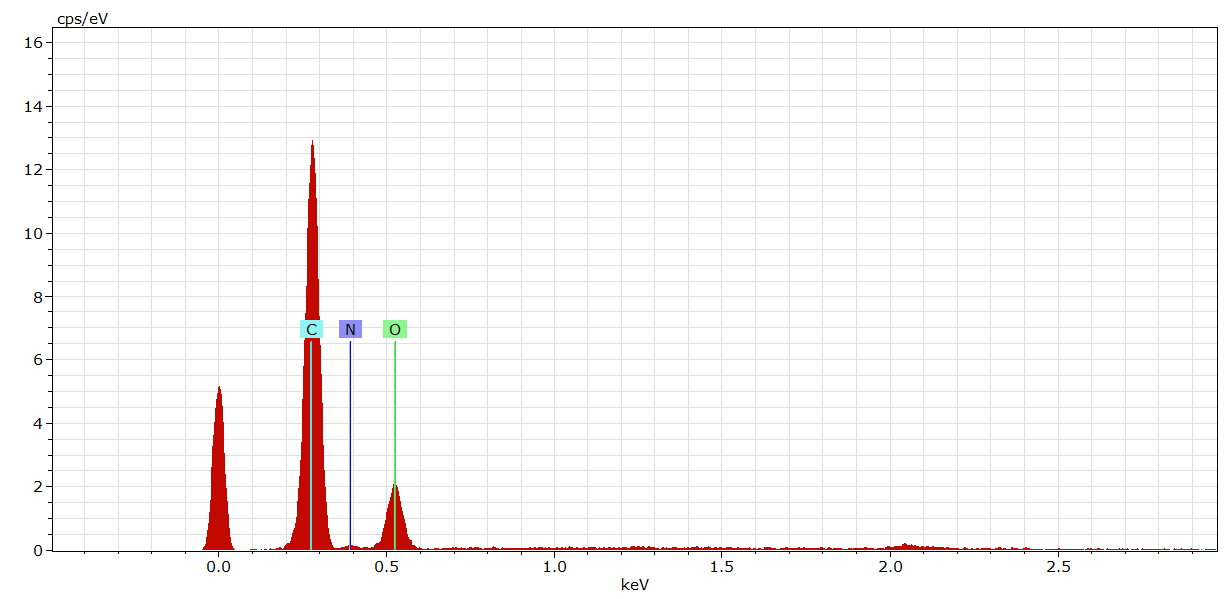


Fig. S4. EDX analysis of AWBC







a

b







c

d

Fig. S5. TEM images of AWBC at (a) 80000, (b) 20000, (c) 8000 (d) 4000 magnifications

Fig. S6. Effect of dose of AWBC on MB adsorption

Fig. S7. Effect of solution pH on MB adsorption

Fig.S8. Effect of initial concentrations as well as reaction temperature on MB adsorption at three temperatures.

Fig. S9. Effect of contact time on MB adsorption


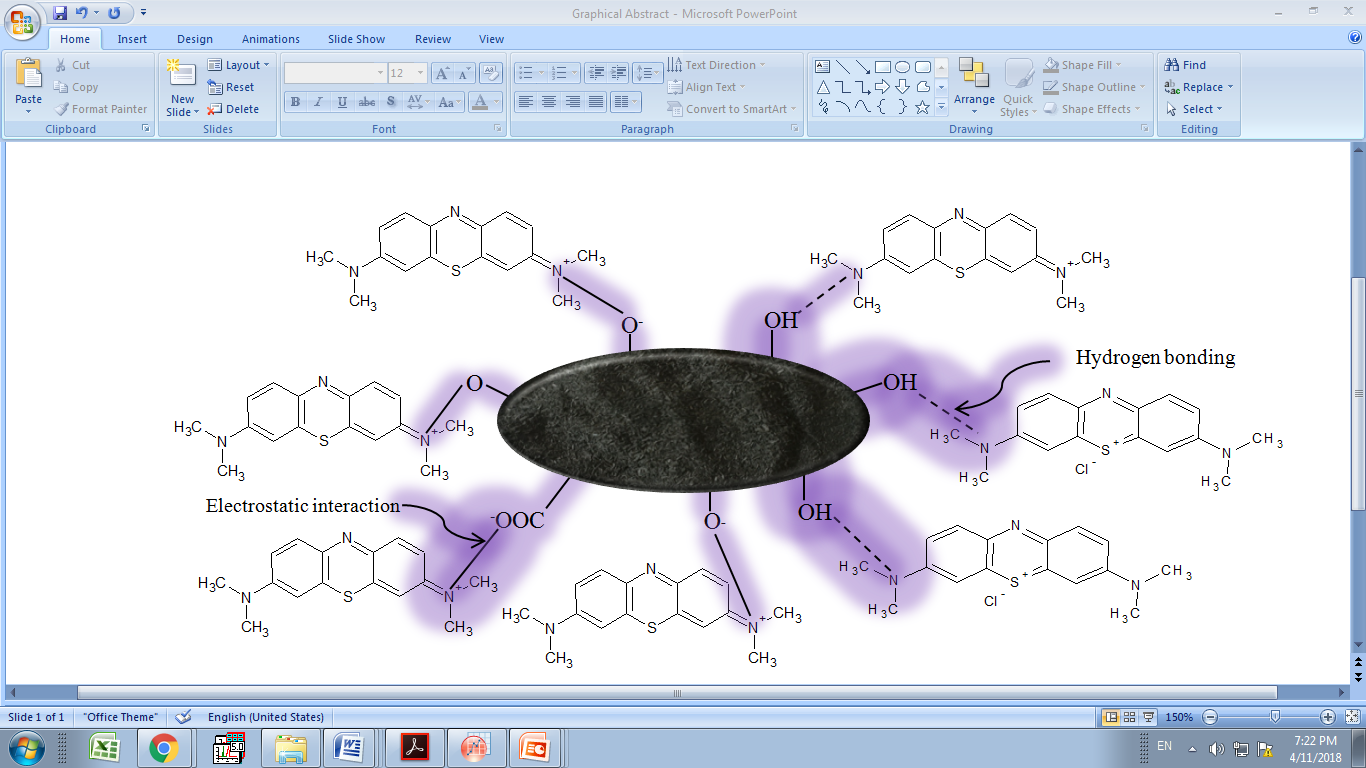


Scheme 1: Proposed mechanistic pathway for electrostatic and hydrogen bonding interactions between MB and AWBC
